# Differential Expression of Major Royal Jelly Proteins in the Hypopharyngeal Glands of the Honeybee *Apis mellifera* upon Bacterial Ingestion

**DOI:** 10.3390/insects13040334

**Published:** 2022-03-29

**Authors:** Yun-Hui Kim, Bo-Yeon Kim, Jin-Myung Kim, Yong-Soo Choi, Man-Young Lee, Kwang-Sik Lee, Byung-Rae Jin

**Affiliations:** 1 College of Natural Resources and Life Science, Dong-A University, Busan 49315, Korea; dbsgml9778@naver.com (Y.-H.K.); boyeon@dau.ac.kr (B.-Y.K.); kjm8578@naver.com (J.-M.K.); 2 Department of Agricultural Biology, National Academy of Agricultural Science, Wanju 55365, Korea; beechoi@korea.kr (Y.-S.C.); mylee33@korea.kr (M.-Y.L.)

**Keywords:** *Apis mellifera*, bacterial challenge, honeybee, major royal jelly protein, vitellogenin

## Abstract

**Simple Summary:**

Transgenerational immune priming (TGIP) to elicit social immunity in the honeybee *Apis mellifera* has two axes: the first is the ingested pathogen fragments–vitellogenin (Vg)–queen’s ovary axis for the developing embryo, and the second is the ingested pathogen fragments–Vg–nurse’s hypopharyngeal gland axis for the queen and young larvae through royal jelly. However, the dynamics of the expression of the major royal jelly proteins (MRJPs) in the hypopharyngeal glands of *A. mellifera* nurse bees after bacterial ingestion must be determined to improve our understanding of the second axis of TGIP. In this study, we investigated the expression patterns of *MRJPs 1*–*7* and *defensin-1* in the hypopharyngeal glands and *Vg* in the fat body of nurse bees fed with live or heat-killed *Paenibacillus larvae* over 12 h or 24 h by using northern blot analysis. We found that the expression of *MRJPs* and *defensin-1* in the hypopharyngeal glands and *Vg* in the fat body was significantly induced in nurse bees upon bacterial ingestion, indicating that the differential expression patterns of *MRJPs*, *defensin-1*, and *Vg* were dependent on the bacterial status and timing of bacterial ingestion. We also found that antimicrobial peptide (*AMP*) genes showed induced expression in young larvae upon bacterial ingestion. In summary, our findings indicate that *MRJPs* in the hypopharyngeal glands are upregulated along with *Vg* in the fat body of nurse bees upon bacterial ingestion, providing novel insights into the ingested pathogen fragments–Vg–nurse’s hypopharyngeal gland axis for TGIP.

**Abstract:**

Honeybee vitellogenin (Vg) transports pathogen fragments from the gut to the hypopharyngeal glands and is also used by nurse bees to synthesize royal jelly (RJ), which serves as a vehicle for transferring pathogen fragments to the queen and young larvae. The proteomic profile of RJ from bacterial-challenged and control colonies was compared using mass spectrometry; however, the expression changes of major royal jelly proteins (MRJPs) in hypopharyngeal glands of the honeybee *Apis mellifera* in response to bacterial ingestion is not well-characterized. In this study, we investigated the expression patterns of *Vg* in the fat body and *MRJPs 1–7* in the hypopharyngeal glands of nurse bees after feeding them live or heat-killed *Paenibacillus larvae*. The expression levels of *MRJPs* and *defensin-1* in the hypopharyngeal glands were upregulated along with *Vg* in the fat body of nurse bees fed with live or heat-killed *P. larvae* over 12 h or 24 h. We observed that the expression patterns of *MRJPs* and *defensin-1* in the hypopharyngeal glands and *Vg* in the fat body of nurse bees upon bacterial ingestion were differentially expressed depending on the bacterial status and the time since bacterial ingestion. In addition, the *AMP* genes had increased expression in young larvae fed heat-killed *P. larvae*. Thus, our findings indicate that bacterial ingestion upregulates the transcriptional expression of *MRJPs* in the hypopharyngeal glands as well as *Vg* in the fat body of *A. mellifera* nurse bees.

## 1. Introduction

Royal jelly (RJ), which is secreted from the hypopharyngeal glands of young nurse honeybees, is the exclusive nourishment for the queen and young larvae. RJ is composed of water, carbohydrates, lipids, proteins, vitamins, mineral, and other components [1]. The major protein components of RJ are major royal jelly proteins (MRJPs), ranging between 83% and 90% of protein components [2,3]. Nine MRJPs in the honeybee *Apis mellifera* have been identified: MRJPs 1–7 in RJ [1,4,5,6,7,8], MRJPs 1–9 in the spermathecal fluid of the honeybee queen [9], and MRJPs 8 and 9 in bee venom [10,11,12,13]. MRJPs 1–7 exhibit antimicrobial and antioxidant activities [14,15,16,17,18,19] as well as the modulation of immune responses [20].

Vitellogenin (Vg), an egg-yolk precursor in insects, is expressed in the fat body, secreted into the hemolymph, and then taken up by developing oocytes [21,22]. The fat body is the primary site of synthesis of Vg and antimicrobial peptides (AMPs) [23,24]. In addition to reproduction, Vg plays a role in immunity, life span, and antioxidation [25,26,27,28,29]. In honeybees, Vg is also taken up in the hypopharyngeal glands of nurses and is used as an amino acid donor for the synthesis of RJ [30,31]. In addition to the delivery of pathogen fragments into the queen’s ovaries by Vg [27], honeybee Vg transports pathogen fragments from the gut to the hypopharyngeal glands, which then results in the incorporation of pathogen fragments into RJ and the subsequent transfer of pathogen fragments to the queen and young larvae [32,33]. Earlier studies have shown that Vg is a transporter in transferring ingested pathogen fragments to the queen’s ovaries and nurse’s hypopharyngeal glands for transgenerational immunity in *A. mellifera* [27,32,33].

Transgenerational immune priming (TGIP) is attracting considerable attention as a means for forming social immunity in honeybees [27,33,34]. In the delivery for TGIP in *A. mellifera*, Vg plays an important role in the transport of ingested pathogen fragments to the queen’s ovaries or the nurse’s hypopharyngeal glands [27,32], and RJ acts as a vehicle in transferring pathogen fragments between nestmates [33]. Most recently, a study involving mass spectrometry reported changes of proteomic profile, including MRJPs 1–7 and defensin-1, in RJ from bacterial-challenged and control colonies [33]. However, the gene expression pattern of *Vg* in the fat body and *MRJPs 1–7* in the hypopharyngeal glands of *A. mellifera* nurse bees upon live or heat-killed bacterial challenge remains unclear. Furthermore, the differential expression of *MRJPs 1–7* and *Vg* in nurse bees fed with live or heat-killed bacteria is yet to be reported, and it is unclear whether the expression pattern of *MRJPs* in hypopharyngeal glands is similar to that of *Vg* in the fat body of nurse bees upon bacterial ingestion. Therefore, the gene expression pattern of *MRJPs* in the hypopharyngeal glands and *Vg* in the fat body of nurse bees must be determined to understand the response of *MRJPs 1–7* in the hypopharyngeal glands of *A. mellifera* nurse bees to bacterial ingestion.

In this study, we performed studies on the transcriptional expression patterns of *MRJPs 1–7* in the hypopharyngeal glands and *Vg* in the fat body of nurse bees fed with live or heat-killed *Paenibacillus larvae*. Here, we report the differential expression of *MRJPs* in the hypopharyngeal glands of *A. mellifera* nurse bees upon bacterial ingestion.

## 2. Materials and Methods

### 2.1. Honeybees

The honeybees (*Apis mellifera*) used in this experiment were reared in an apiary at Dong-A University. Newly emerged workers (1-day-old) were marked on the thorax with a spot of paint marker (Monami Co., Ltd., Seoul, Korea) and introduced into a colony. The marked worker bees were collected from the colony when they were six days old.

### 2.2. Feeding Experiment

Six-day-old *A. mellifera* nurse bees were placed in cages (11.3 × 7 × 4.3 cm) with 40% sucrose solution and bee bread. American foulbrood *P. larvae* [35] was cultured in Luria–Bertani (LB) medium [36]. The heat treatment of *P. larvae* was autoclaved twice at 121 °C for 15 min. The resulting non-viable *P. larvae* cells were confirmed by culture on LB agar plates. The dose of *P. larvae* used in this study was determined based on previous studies [36,37]. The nurse bees were fed ad libitum a diet without or with live or heat-killed *P. larvae* (1 × 10^5^ cells per nurse bee) over 12 h or 24 h (*n* = 7 for each treatment). The cages were incubated in an incubator at 34 °C with 80% humidity. After incubation over 12 h or 24 h, *A. mellifera* nurse bees were dissected on ice under a stereo-microscope (Zeiss, Jena, Germany). Tissue samples (fat body and hypopharyngeal glands) from *A. mellifera* nurse bees were individually collected and washed twice with phosphate-buffered saline (PBS; 140 mM NaCl, 27 mM KCl, 8 mM Na_2_HPO_4_, 1.5 mM KH_2_PO_4_, pH 7.4). For the bacterial ingestion to young larvae of *A. mellifera*, eggs were collected from the same colony. The dietary composition and amount of larval diet were prepared according to the in vitro rearing protocol of honeybee workers [38]. The young larvae (1 day old), which receive RJ from nurse bees during the first day of the larval stage [39], were supplied with a larval diet (royal jelly 44.25%, glucose 5.30%, fructose 5.30%, yeast extract 0.90%, and water 44.25%) [38] without or with heat-killed *P. larvae* (1 × 10^5^ cells per larva) in 96-well plates. After feeding in an incubator at 34 °C with 80% humidity over 24 h or 48 h, the young larvae were individually collected (*n* = 9 for each treatment).

### 2.3. RNA Extraction

Total RNA was directly extracted from the fat body and hypopharyngeal glands that were freshly dissected from *A. mellifera* nurse bees by using TRIzol reagent (Invitrogen, Carlsbad, CA, USA), as per the manufacturer’s protocol. Total RNA from young larvae of *A. mellifera* was extracted from the whole body.

### 2.4. Northern Blot Analysis

Total RNA extracted from the fat body, hypopharyngeal glands, and whole body of *A. mellifera* was subjected to electrophoresis on a 1.0% formaldehyde agarose gel (5 µg of total RNA/lane). After electrophoresis, total RNA was transferred onto a nylon membrane (Schleicher & Schuell, Dasell, Germany). The cDNA probes were labeled with [α-^32^P] dCTP (Amersham Biosciences, Piscataway, NJ, USA) using a Prime-It II Random Primer Labeling Kit (Stratagene, La Jolla, CA, USA) as per the manufacturer’s protocol. The following cDNAs for probes of northern blot were used: *Vg* (NM_001011578), *MRJPs 1–7* (NM_001011579, NM_001011580, NM_001011601, NM_001011610, NM_001011599, NM_001011622, and NM_001014429), *defensin-1* (NM_001011616), *abaecin* (NM_001011617), *hymenoptaecin* (NM_001011615), and *ß-actin* (AB023025). Hybridization and exposure were performed as described by Park et al. [29]. The signal intensities were analyzed using AlphaEaseFC (VER. 4.0), a computerized image-analysis system (Alpha Innotech Co., San Leandro, CA, USA). Data are presented the mean ± standard deviation (SD) of signals from three measurements.

### 2.5. Statistical Analysis

To determine the statistical significance at *p*-values < 0.05, the data were analyzed using one-way ANOVA with post hoc Tukey’s test (Statistical software SPSS version 22.0, IBM Inc., Chicago, IL, USA).

## 3. Results

### 3.1. Expression Profile of Vg and Abaecin in the Fat Body of Nurse Bees

**Figure 1:**
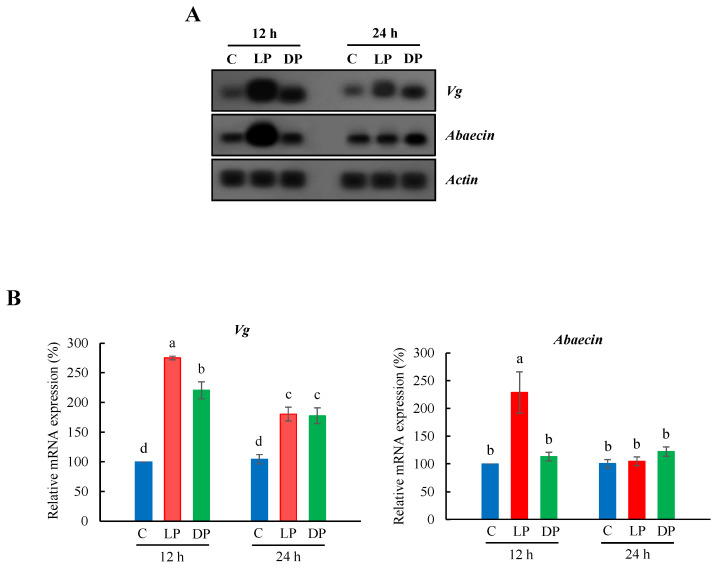
Expression profile of *Vg* and *abaecin* in the fat body of *A. mellifera* nurse bees fed with live or heat-killed *P. larvae*. (**A**) The expression of *Vg* and *abaecin*, as ascertained by northern blot analysis (*n* = 7). *β-Actin* was used as an internal control to depict the total RNA loading amount. C, untreated controls; LP, feeding with live *P. larvae*; DP, feeding with heat-killed *P. larvae*. 12 h and 24 h represent feeding time. (**B**) The relative levels of *Vg* and *abaecin* mRNAs represent the average band densities of the target genes normalized to the expression levels of the control (12 h). The bars represent the mean ± SD from three measurements. A one-way ANOVA test was used to determine the significant difference (*p* < 0.05) with different lowercase letters (a–d).

Gene expression of *Vg* and *abaecin* in the fat body of nurse bees fed with live or heat-killed *P. larvae* over 12 h or 24 h was examined (Figure 1A). Gene expression levels of *Vg* increased substantially in the fat body of nurse bees fed with live or heat-killed *P. larvae* over 12 h or 24 h (Figure 1B). Ingestion of live *P. larvae* over 12 h had a higher impact on *Vg* expression compared to heat-killed *P. larvae*, whereas bacterial ingestion over 24 h showed a similar increase in Vg expression in the fat body of nurse bees fed with live and heat-killed *P. larvae*. *Abaecin* expression was highly upregulated in feeding with live *P. larvae* over 12 h and showed a significant increase in feeding with heat-killed *P. larvae* over 24 h (Figure 1B).

### 3.2. Expression Profile of MRJPs and Defensin-1 in Hypopharyngeal Glands of Nurse Bees

**Figure 2:**
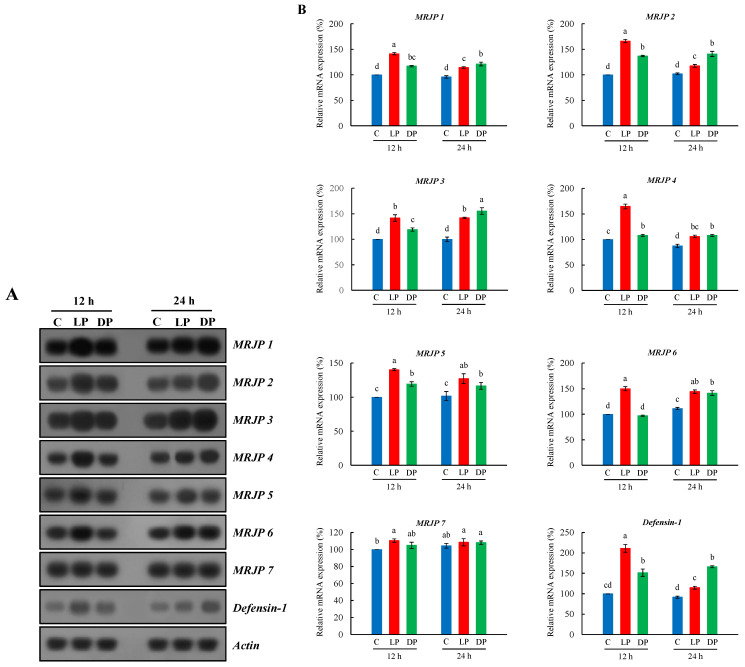
Expression profile of *MRJPs 1–7* and *defensin-1* in hypopharyngeal glands of *A. mellifera* nurse bees fed with live or heat-killed *P. larvae*. (**A**) The expression of *MRJPs 1–7* and *defensin-1*, as ascertained by northern blot analysis (*n* = 7). *β-Actin* was used as an internal control to depict the total RNA loading amount. C, untreated controls; LP, feeding with live *P. larvae*; DP, feeding with heat-killed *P. larvae*. 12 h and 24 h represent feeding time. (**B**) The relative levels of *MRJPs 1–7* and *defensin-1* mRNAs represent the average band densities of the target genes normalized to the expression levels of the controls (12 h). The bars represent the mean ± SD from three measurements. A one-way ANOVA test was used to determine the significant difference (*p* < 0.05) with different lowercase letters (a–d).

The gene expression of *MRJPs* (*MRJP1* through *MRJP 7*) and *defensin-1* in the hypopharyngeal glands of nurse bees fed with live or heat-killed *P. larvae* over 12 h or 24 h was investigated (Figure 2A). We found that the expression patterns of *MRJPs* and *defensin-1* in the hypopharyngeal glands of nurse bees were highly upregulated in feeding with live or heat-killed *P. larvae* over 12 h or 24 h (Figure 2B). *MRJPs* and *defensin-1* in hypopharyngeal glands of nurse bees fed with live *P. larvae* over 12 h showed a higher expression compared to heat-killed *P. larvae*. However, bacterial ingestion over 24 h demonstrated more impact on the expression of *MRJPs* and *defensin-1* in feeding with heat-killed *P. larvae* compared to live *P. larvae* (Figure 2B). In the result of expression profile of *MRJPs*, no clear difference in *MRJP 7* expression between bacterial ingestion and control was observed. *Defensin-1* expression pattern was similar to the expression of *MRJPs*.

### 3.3. Expression Profile of Defensin-1, Hymenoptaecin, and Abaecin in Whole Body of Young Larvae

**Figure 3:**
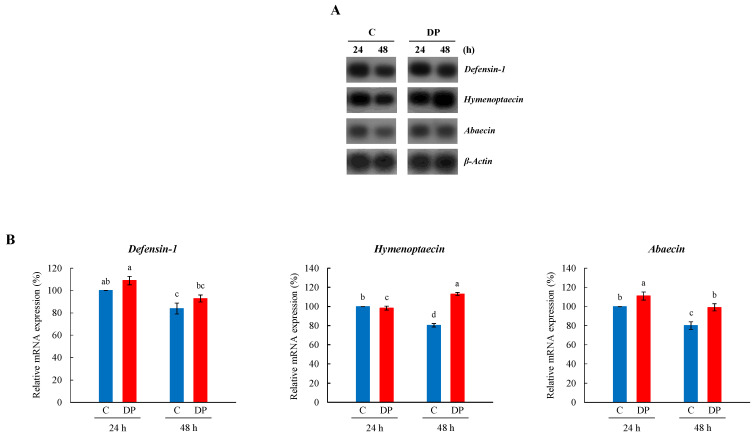
Expression profile of *defensin-1*, *hymenoptaecin*, and *abaecin* in the whole body of *A. mellifera* young larvae fed with heat-killed *P. larvae*. (**A**) The expression of *defensin-1*, *hymenoptaecin*, and *abaecin*, as ascertained by northern blot analysis (*n* = 9). *β-Actin* was used as an internal control to depict the total RNA loading amount. C, untreated controls; DP, feeding with heat-killed *P. larvae*. 24 h and 48 h represent feeding time. (**B**) The relative levels of *defensin-1*, *hymenoptaecin*, and *abaecin* mRNAs represent the average band densities of the target genes normalized to the expression levels of the control (24 h). The bars represent the mean ± SD from three measurements. A one-way ANOVA test was used to determine the significant difference (*p* < 0.05) with different lowercase letters (a–d).

Gene expression of *AMP* (*defensin-1*, *hymenoptaecin*, and *abaecin*) in the whole body of young larvae fed with heat-killed *P. larvae* over 24 h or 48 h was examined (Figure 3A). The gene expression patterns of *defensin-1*, *hymenoptaecin*, and *abaecin* showed a tendency of significant differences between heat-killed *P. larvae* ingested and control young larvae: *AMP* expression showed a significant increase in *defensin-1* in feeding over 24 h, *hymenoptaecin* in feeding over 48 h, and *abaecin* in feeding over both 24 h and 48 h.

## 4. Discussion

TGIP seems to be an effective way to elicit social immunity in honeybees [27,32,33,34]. The model representing TGIP in *A. mellifera* [27,32,33] is known to have two axes: one is the ingested pathogen fragments–Vg–queen’s ovary axis to elicit an immune response in the developing embryo, and the other is the ingested pathogen fragments–Vg–nurse’s hypopharyngeal gland axis to elicit an immune response in the queen and young larvae through RJ. In these axes, Vg and RJ play a role as transporters [27,32,33]. As Vg, which is transferred through ingested bacterial fragments to the hypopharyngeal glands of nurse bees [32,33], is used as an amino acid donor for synthesis of RJ [30,31] and MRJPs are major protein components of RJ [2,3], we focused on the expression patterns of *MRJPs* in the hypopharyngeal glands of nurse bees upon bacterial ingestion.

As Vg showed induced expression in the fat body of bees against bacterial challenge [29] and was bound to bacterial pathogens, which are transferred by Vg to the queen’s ovaries or nurse’s hypopharyngeal glands [27,32], first, we determined the expression pattern of *Vg* in nurse bees upon bacterial ingestion. We found that *Vg* showed relatively high induction in the fat body of nurse bees fed with live *P. larvae* over 12 h, whereas *Vg* expression in feeding over 24 h increased in a similar manner between live and heat-killed *P. larvae*. These results revealed that *Vg* is highly upregulated along with *abaecin* upon bacterial ingestion, which indicates that Vg is involved in the innate immunity of *A. mellifera* nurse bees.

A recent study applying mass spectrometry reported that the relative protein abundance in RJ from bacterial-challenged and control colonies exhibited minimal changes, and defensin-1 showed modest increases in RJ from pathogen-diet colonies compared to controls [33]. Another study showed that *defensin-1* is not induced in honeybee adults after a colony-level *P. larvae* infection [40]. In this study, our results point to changes in the gene expression patterns of *MRJPs* and *defensin-1* in the hypopharyngeal glands of nurse bees in response to bacterial ingestion. Notably, the expression profiles of *MRJPs 1–6* and *defensin-1* in the hypopharyngeal glands of nurse bees were highly upregulated after feeding with live *P. larvae* over 12 h and feeding with heat-killed *P. larvae* over 24 h. Furthermore, the expression patterns of *MRJPs* in the hypopharyngeal glands and *Vg* in the fat body of nurse bees indicated that *Vg* and *MRJPs* were differentially expressed, depending on the exposure to live or heat-killed *P. larvae*. Thus, our results suggest that the ingestion of live *P. larvae* rapidly induced the expression of *MRJPs*, *AMPs*, and *Vg* in nurse bees compared to the ingestion of heat-killed *P. larvae*, whereas the ingestion of heat-killed *P. larvae* seemed to have a relatively long-lasting effect on the regulation of the expression of *MRJPs*, *AMPs*, and *Vg*. Considering that ingested bacteria are transferred by Vg to the hypopharyngeal glands of nurse bees, and then they are transported into RJ [32,33], it is likely that upon bacterial ingestion, the increased level of *Vg* expression in the fat body leads to increased *MRJPs* and *defensin-1* in the hypopharyngeal glands of nurse bees. This finding suggests an association between the expression patterns of *MRJPs* and *defensin-1* in the hypopharyngeal glands and *Vg* in the fat body of nurse bees, depending on the bacterial status and the time since bacterial ingestion. Considering that the gene expression of *abaecin*, *defensin-1*, and *hymenoptaecin* in bumblebees showed high levels beginning at 8 h until 12 h post injection of live bacteria, and then decreased slightly 24 h post injection [41], our results confirmed that the differential expression level of *MRJPs* and *defensin-1* in hypopharyngeal glands of nurse bees fed with live or heat-killed *P. larvae* over 12 h or 24 h was dependent on the bacterial status. We have recently demonstrated that MRJPs bind to the cell walls of bacteria and exhibit antimicrobial activities at different levels [18]. Thus, our findings suggest that the increased expression levels of *MRJPs* and *defensin-1* in the hypopharyngeal glands of nurse bees upon bacterial ingestion represent an immune response of MRJPs in RJ of *A. mellifera*. However, in the present study, we were unable to observe a clear difference in the *MRJP 7* expression profile between the bacterial ingestion group and the control. Additional studies will be required to determine the role of upregulated MRJPs in the hypopharyngeal glands of nurse bees after bacterial ingestion.

The second axis in TGIP is that the ingested bacteria are transferred by Vg to the hypopharyngeal glands of nurse bees and transported into RJ, which then elicits an immune response in the queen and young larvae through RJ [32,33]. We examined the gene expression patterns of *AMPs* in *A. mellifera* young larvae fed with heat-killed *P. larvae* and found that *AMPs* are differentially induced in young larvae upon bacterial ingestion: *defensin-1* in feeding over 24 h, *hymenoptaecin* in feeding over 48 h, and *abaecin* in feeding over 24 h and 48 h. The results of this study indicate that although *AMPs*, especially in *defensin-1*, are induced at a modest level, *AMPs* show differential expression in young larvae of *A. mellifera* in response to bacterial ingestion. Moreover, the expression profile of *AMPs* in young larvae indicates that bacterial ingestion during the first day of the larval stage has immune priming effects on the second (24 h) and third (48 h) days of *A. mellifera* young larval stage.

## 5. Conclusions

Our findings provide the first evidence that *MRJPs* in the hypopharyngeal glands of nurse bees fed with live or heat-killed *P. larvae* were differentially expressed depending on bacterial status and time of bacterial ingestion. The present study indicates that the expression of *MRJPs* and *defensin-1* in hypopharyngeal glands shows a pattern similar to the *Vg* expression in the fat body of nurse bees upon bacterial ingestion. These findings show the differential expression of *MRJPs* in the hypopharyngeal glands of nurse bees in response to bacterial ingestion, providing novel insights into MRJPs to better understand the ingested pathogen fragments–Vg–nurse’s hypopharyngeal gland axis for TGIP.

## Data Availability

The data presented in this study are available in the article.
